# Lyophilized Maqui (*Aristotelia chilensis*) Berry Administration Suppresses High-Fat Diet-Induced Liver Lipogenesis through the Induction of the Nuclear Corepressor SMILE

**DOI:** 10.3390/antiox10050637

**Published:** 2021-04-21

**Authors:** Viviana Sandoval, Hèctor Sanz-Lamora, Pedro F. Marrero, Joana Relat, Diego Haro

**Affiliations:** 1 Escuela de Nutrición y Dietética, Facultad de Ciencias para el Cuidado de la Salud, Universidad San Sebastián, Sede De la Patagonia, Puerto-Montt 5501842, Chile; vsandovals@docente.uss.cl; 2 Department of Nutrition, Food Sciences and Gastronomy, School of Pharmacy and Food Sciences, Food Torribera Campus, University of Barcelona, E-08921 Santa Coloma de Gramenet, Spain; h.sanz.lamora@ub.edu (H.S.-L.); pedromarrero@ub.edu (P.F.M.); 3 Institute of Nutrition and Food Safety, University of Barcelona (INSA-UB), E-08921 Santa Coloma de Gramenet, Spain; 4 Institute of Biomedicine, University of Barcelona (IBUB), E-08028 Barcelona, Spain; 5 CIBER Physiopathology of Obesity and Nutrition (CIBER-OBN), Instituto de Salud Carlos III, E-28029 Madrid, Spain

**Keywords:** anthocyanins, nonalcoholic liver disease, SMILE, high-fat diet, maqui berry, lipogenesis, fatty acid oxidation

## Abstract

The liver is one of the first organs affected by accumulated ectopic lipids. Increased de novo lipogenesis and excessive triglyceride accumulation in the liver are hallmarks of nonalcoholic fatty liver disease (NAFLD) and are strongly associated with obesity, insulin resistance, and type 2 diabetes. Maqui dietary supplemented diet-induced obese mice showed better insulin response and decreased weight gain. We previously described that these positive effects of maqui are partially due to an induction of a brown-like phenotype in subcutaneous white adipose tissue that correlated with a differential expression of *Chrebp* target genes. In this work, we aimed to deepen the molecular mechanisms underlying the impact of maqui on the onset and development of the obese phenotype and insulin resistance focusing on liver metabolism. Our results showed that maqui supplementation decreased hepatic steatosis caused by a high-fat diet. Changes in the metabolic profile include a downregulation of the lipogenic liver X receptor (LXR) target genes and of fatty acid oxidation gene expression together with an increase in the expression of *small heterodimer partner interacting leucine zipper protein* (*Smile*), a corepressor of the nuclear receptor family. Our data suggest that maqui supplementation regulates lipid handling in liver to counteract the metabolic impact of a high-fat diet.

## 1. Introduction

The regular consumption of anthocyanins and anthocyanidin-rich berries is considered a potential strategy for the treatment/prevention of obesity-related pathologies, among others [1,2,3,4,5,6,7,8,9]. The intake of anthocyanins or anthocyanin-rich foods has shown positive effects for bodyweight control both in humans and in rodents. It has also proven effective for reducing fat accumulation, improving glucose tolerance, improving insulin sensitivity, and increasing energy expenditure, inter alia [10,11,12,13,14,15,16,17,18]. However, there is little information about the molecular mechanisms underlying these effects.

The liver is a key organ in the maintenance of metabolic homeostasis and is one of the first organs affected by accumulated ectopic lipids. Two of the hallmarks of nonalcoholic fatty liver disease (NAFLD) are its higher rate of de novo lipogenesis and its excessive accumulation of triglycerides in the liver. These two parameters are strongly associated with obesity and insulin resistance, as well as type 2 diabetes [19,20]. The accumulation of intrahepatic fat that leads to liver steatosis is now recognized as the hepatic manifestation of metabolic syndrome and a factor responsible for the metabolic complications associated with obesity [21].

Maqui (*Aristotelia chilensis*) is a berry from Chile with a characteristic profile of anthocyanins, where the main representatives are delphinidin-3-*O-*sambubioside-5-*O*-glucoside and delphinidin-3-*O*-sambubioside [22,23]. The consumption of maqui has shown antioxidant effects and a beneficial impact on fasting glucose and insulin levels both in humans and in rodent models of obesity and type 2 diabetes [24,25,26,27]. The hypoglycemic activity of maqui has been linked to delphinidin-3-sambubioside-5-glucoside, which has been pointed out as the molecule responsible for this effect in vivo [25].

In our previous work, we provided evidence that a dietary supplementation with maqui ameliorated part of the unhealthy effects caused by a high-fat diet (HFD) [23]. The published results demonstrated that, in mice, maqui administration induces the expression of fuel storage and thermogenic genes, giving to the subcutaneous white adipose tissue (scWAT) a brown-like phenotype. Our data suggested that maqui could exert its effects, at least in part, through the upregulation of carbohydrate responsive element binding protein b (*Chrebpb*) expression and improved fibroblast growth factor 21 (FGF21) signaling.

With the global aim of describing the molecular mechanisms underlying the metabolic effects of maqui, we evaluated the impact of lyophilized maqui when added to beverages, focusing specifically on the progression of diet-induced obesity (DIO). The liver metabolic profile and hepatic steatosis were analyzed in mice subjected to a HFD for 16 weeks supplemented or not with maqui.

We showed that maqui supplementation decreased hepatic steatosis and the triglyceride accumulation caused by a HFD. This change in liver lipid content observed in maqui-supplemented animals correlates with a downregulation in the expression of fatty acid oxidation genes and an upregulation in the expression of the nuclear receptor family’s corepressor SMILE (small heterodimer partner interacting leucine zipper protein), which coincides with a downregulation of the nuclear receptor LXR (lipogenic liver X receptor) target genes. SMILE belongs to the basic leucine zipper family transcription factors and functions as a nuclear corepressor of several members of the nuclear receptor family [28]. Among others, it has been shown that SMILE negatively regulates LXR alfa transcriptional activity by directly interacting with it and competing with its coactivator SRC-1. Moreover, SMILE overexpression inhibits LXR alfa-mediated gene expression of the sterol regulatory binding protein 1c (*Srebp-1c*) and decreased hepatic LXR alfa agonist-induced triglycerides (TG) and lipid accumulation [29].

## 2. Materials and Methods

### 2.1. Animal Procedures: Dosage Regimen

All animal procedures used in this article were approved by the Animal Ethics Committee of the University of Barcelona (CEEA-137/18) and were previously described in Sandoval et al. [23]. Briefly, 4-week-old C57BL/6J male mice (*n* = 23) were housed in a temperature-controlled room (22 ± 1 °C) under a 12-h/12-h light/dark cycle with free access to filtered tap water and a rodent chow diet. Before the initiation of the nutritional intervention, animals were tested as normoglycemic and randomly assigned into two groups: (1) HFD (*n* = 9) and (2) HFDM, HFD supplemented with maqui (*n* = 14). Both experimental groups were fed a HFD containing 45% of calories from fat and 4.73 kcal per gram of food (D12451, Research Diets) for 16 weeks with free access to food and water. Moreover, HFDM mice were supplemented with 4 mg/day of lyophilized maqui (Maqui berry, Native for Life, Chile) added to filtered tap water (20 mg of lyophilized maqui/mL of filtered tap water). To prevent the oxidation of bioactive compounds, the maqui-supplemented water was prepared every two days. The calculation of the maqui’s dosage regimen was based on the polyphenol intake recommended as beneficial by the PREDIMED study (820 mg in a human diet of 2300 kcal) [30,31]. The nutritional information of the lyophilized maqui berry and details of the dosage regimen are detailed in Sandoval et al. [23].

During the nutritional intervention period, the intake of food and beverages was recorded every two days and bodyweight progression was evaluated twice a week. The intake of food and water was measured by the difference between what we put in the cage and what we recovered two days later. The results of the food and liquid intake, including the calorie intake and the bodyweight progression, were published in [23], where it was shown that HFDM mice displayed a lower bodyweight increase after 16 weeks of nutritional intervention, even if they exhibited a higher calorie intake. At week 16, the animals were euthanized. Blood was extracted via an intracardiac puncture, and serum was obtained via centrifugation (1500 rpm, 20 min). The tissues as liver, heart, epididymal, and subcutaneous fat and brown fat were isolated, immediately snap-frozen, and stored at −80 °C for future analysis.

### 2.2. Blood Baseline Glucose Levels

Blood samples were collected from the tail vein and glucose levels were measured using a glucometer (Glucocard SM, Menarini, Florence, Italy). These measurements were performed on week 15, where mice were previously fasted for 6 h in the morning.

### 2.3. Liver Triglyceride Content

Liver tissue (100 mg) of each mouse was homogenized in a 1 mL solution of 5% Nonidet P40 (A1694,0250, PanReac AppliChem, Barcelona, Spain) with ultrapure water. The amount of triglyceride (TG) was determined using the Triglyceride Quantification Colorimetric Kit (MAK266, Sigma Aldrich, St. Louis, MI, USA).

### 2.4. RNA Isolation and Quantitative PCR Analysis

Total RNA was isolated from frozen liver using TRIzol Reagent™ (15609415, Fisher Scientific, Waltham, MA, USA) and genomic DNA traces were removed with the RapidOut DNA Removal Kit™ (13565150, Thermo Scientific, Waltham, MA, USA). Moreover, 1 μg of total RNA was converted to cDNA using the High-Capacity cDNA Reverse Transcription Kit™ (10400745, Applied Biosystems, Foster City, CA, USA). Relative mRNA levels were measured via quantitative PCR (qPCR) using a SYBR™ Select Master Mix for CFX (13206529, Applied Biosystems™, Foster City, CA, USA). The sequences of the primers (Sigma Aldrich, St. Louis, MI, USA) are shown in Table S1. The relative expression of mRNA was normalized using 18S and B2M as housekeeping genes. The 18S gene was used to normalize genes that were analyzed with TaqMan probes and B2M when SYBR green primers were used. The stability of these genes is shown in Supplemental Figure S1. Results were obtained by the relative standard curve method, and values of the HFD group were set to 1.

### 2.5. Histological Analysis

For the histological analysis, pieces of liver from each animal were fixed in 10% formalin (Ref Sigma Aldrich, St. Louis, MI, USA) and embedded in paraffin. Next, 4 µm-thick sections were cut and stained with hematoxylin and eosin (H&E). The images were acquired in a Digital Upright Microscope BA310 Digital and a Moticam 2500 camera. The selection of test objects was performed according to color and choosing the same limits for the binarization of all images. At least three pictures from different regions of each cut were taken.

### 2.6. Data Analysis/Statistics

Values were expressed as means ± SEM, and a *p*-value of <0.05 was considered statistically significant. Statistical analyses of the data were performed using GraphPad Prism version 8.02 (GraphPad, San Diego, CA, USA). The significance was determined using an unpaired two tailed Student’s *t* test and with Welch’s correction without equal SDs.

## 3. Results

### 3.1. Maqui Administration Decreases Liver Steatosis and TG Content

Our previous published data showed that maqui dietary supplementation of diet-induced obese mice partially counteracted the unhealthy metabolic impact of a HFD by acting in the subcutaneous white adipose tissue (scWAT) [23]. Since hepatic metabolism is also central to energy homeostasis, in this work, we analyzed the impact of lyophilized maqui berry supplementation in the liver of mice subjected to HFD.

**Figure 1:**
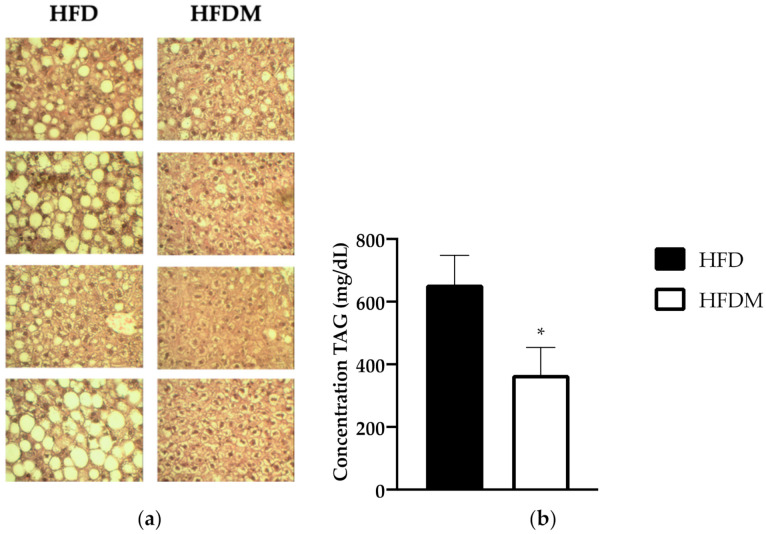
Maqui dietary supplementation ameliorates liver steatosis and decreases triglyceride (TG) content. (**a**) Histologic analysis of liver. Representative pictures of the hematoxylin and eosin (H&E)-stained sections of livers from a high-fat diet (HFD) and maqui supplemented HFD (HFDM) (40× magnification). Large lipid droplets (LDs) were observed in HFD images compared to the HFDM group. (**b**) Hepatic TG content. The concentration of TG (ng/uL) was measured in the livers of HFD (*n* = 9) and HFDM (*n* = 14) mice. Data are presented as the mean ± SEM. * *p* < 0.05.

Histological analysis of livers using eosin–hematoxylin staining revealed that mice undergoing HFD, as expected, exhibited a large accumulation of lipids in this organ, and large lipid droplets (LDs) were observed, thus confirming advanced hepatic steatosis (Figure 1a). In contrast, the liver of diet-induced obese mice supplemented with maqui showed fewer and smaller LDs, indicating that maqui supplementation improves hepatic steatosis caused by a HFD (Figure 1a). According to the histology results, the maqui-supplemented group of mice displayed a concomitant decrease in hepatic TG content (Figure 1b).

Liver is a key organ for maintaining metabolic homeostasis and blood glucose levels through the regulation of fatty acid oxidation and gluconeogenesis. Both pathways are closely related under fasting conditions as fatty acid oxidation is needed to activate gluconeogenesis [32,33,34]. In order to evaluate the hepatic metabolic profile of HFDM mice, we analyzed the expression levels of fatty acid oxidation and gluconeogenic genes.

### 3.2. Fatty Acid Oxidation Gene Expression Is Downregulated by Maqui Consumption within a HFD

**Figure 2:**
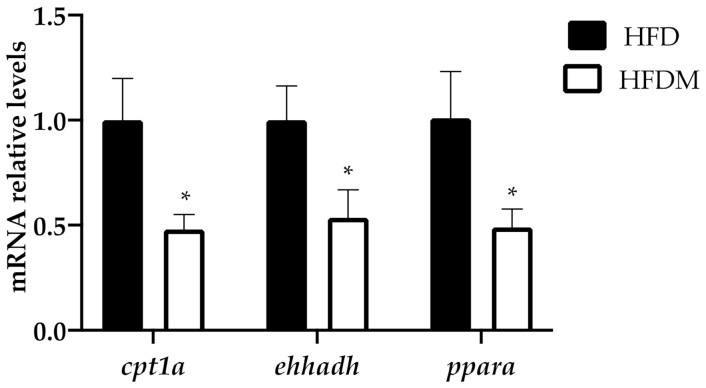
Fatty acid oxidation genes are downregulated in the livers of HFD-fed mice supplemented with maqui. Relative hepatic mRNA levels of *carnitine palmitoyltransferase 1a* (*cpt1a*), *enoyl-CoA hydratase, 3-hydroxyacyl-CoA dehydrogenase (ehhdah),* and *peroxisome proliferator activated receptor alpha (ppara)* were measured via quantitative PCR (qPCR) in HFD (*n* = 9) and HFDM (*n* = 14) animals. Bars represent the relative mRNA levels in the HFD animals, which was the control group and was thus assigned an arbitrary value of 1 in the HFDM. Data are presented as the mean ± SEM. * *p* < 0.05 versus the HFD group.

As hepatic fatty acid oxidation is central to systemic energy balance and liver steatosis susceptibility (associated with peroxisome proliferator-activated receptor alpha (PPARa) dysfunction), we analyzed the mRNA levels of *ppara* and the fatty acid oxidation-related PPARa target genes. Figure 2 shows the expression of *ppara, carnitine palmitoyl transferase 1 (cpt1a)*, the key enzyme in the carnitine-dependent transport of long-chain fatty acids across the mitochondrial inner membrane, and *enoyl-coenzyme A, hydratase/3-hydroxyacyl coenzyme A dehydrogenase* (*ehhadh)*, one of the key enzymes in the peroxisomal beta-oxidation pathway. The three genes are downregulated in maqui-supplemented HFD-fed mice, indicating a reduction in the oxidative capacity of long and very long chain fatty acids compared to the HFD-fed animals.

### 3.3. The Gluconeogenic Gene g6Pase Expression Decreases after Maqui Supplementation

The impairment of hepatic glucose production is considered a hallmark of insulin resistance and a key point for its treatment [35,36]. Moreover, as has been mentioned before, the activation of the gluconeogenic pathway depends on the fatty acid oxidation rate, which is downregulated in HFDM mice (Figure 2).

**Figure 3:**
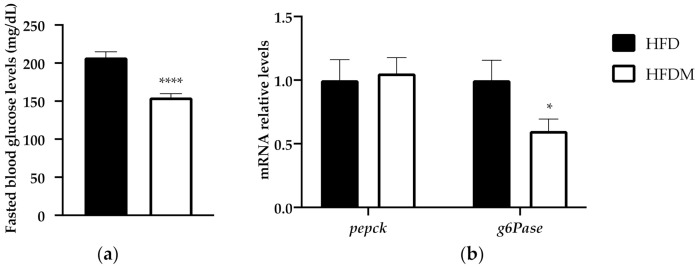
Maqui supplementation reduces fasting blood glucose and gluconeogenesis. (**a**) The fasting blood levels of glucose were measured at week 15 of the nutritional intervention in 6 h-fasted animals. (**b**) Relative hepatic mRNA levels of *phosphoenolpyruvate carboxykinase (pepck)* and *glucose-6-phosphate (g6Pase)* were measured via qPCR in HFD (*n* = 9) and HFDM (*n* = 14) animals. Bars represent the relative mRNA levels in the HFD animals, which was the control group and was thus assigned an arbitrary value of 1 in the HFDM. Data are presented as the mean ± SEM. * *p* < 0.05 versus the HFD group; **** *p* < 0.0001 versus the HFD group.

In order to go deep into the metabolic modifications elicited via maqui supplementation, we analyzed the blood glucose levels in 6 h-fasted mice. The expressions of *phosphoenolpyruvate carboxykinase (pepck)* and *glucose-6-phosphatase (g6Pase)* were the key enzymes in hepatic glucose production in fed animals. Fasting glucose levels (Figure 3a) and the expression of *g6Pase,* but not *pepck* (Figure 3b), decreased in the maqui-supplemented mice.

The reduction in the fasting glucose levels and in the downregulation of *g6Pase* is in accordance with the previous published data where maqui supplementation improved glucose tolerance in HFD-fed mice [23].

### 3.4. The Expression of the Nuclear Receptor Corepressor Smile Increased under Maqui Supplementation

SMILE and peroxisome proliferator-activated receptor gamma coactivator 1 alpha (PGC1a) are both regulators of gluconeogenesis because they control the expression of key gluconeogenic genes. SMILE is an insulin-inducible corepressor that suppresses hepatic gluconeogenesis [37], while PGC1a acts as a coactivator of gluconeogenic genes. Accordingly, with its effect on gluconeogenesis, maqui supplementation induces *smile* gene expression (Figure 3) and diminishes *pgc1a* gene expression (Figure 3). These data correlate with the expression profile of *g6Pase*, as shown in Figure 3b.

### 3.5. Hepatic Lipogenic Gene Expression Is Downregulated by Maqui Supplementation

SMILE has been described as a corepressor of the nuclear receptor family and has been identified as an inhibitor of the transcriptional activity of LXRa and LXRa-mediated SREBP1c gene expression [29]. To confirm the role of SMILE on the hepatic metabolic effects of maqui, we analyzed the expression of LXR and LXRa target genes, including the key enzymes of the lipogenic pathway.

**Figure 4:**
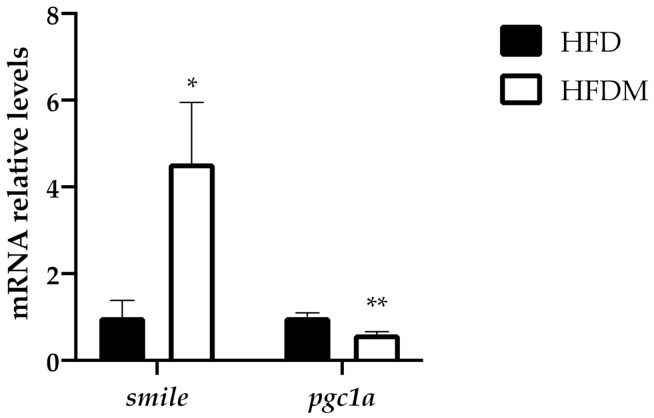
Maqui supplementation upregulates the expression of smile and downregulates pgc1a levels. Relative hepatic mRNA levels of small heterodimer partner interacting leucine zipper protein (smile) and peroxisome proliferator-activated receptor gamma coactivator 1 alpha (pgc1a) were measured via qPCR in HFD (n = 9) and HFDM (n = 14) animals. Bars represent the relative mRNA levels in the HFD animals, which was the control group and was thus assigned to an arbitrary value of 1 in the HFDM group. Data are presented as the mean ± SEM. * *p* < 0.05 versus the HFD group; ** *p* < 0.01 versus the HFD group.

Although neither the expression of *lxra* nor *lxrb* were changed, the expression of the LXRa target genes *cholesterol 7 alpha-hydroxylase* (*cyp7a1*) and *phospholipid transfer protein (pltp)* was downregulated in HFDM mice (Figure 4). In accordance with these results, which indicated a blockade of LXR transcriptional activity, the mRNA levels of *fatty acid synthase (fasn), stearoyl-CoA desaturase-1 (scd1), fatty acid elongase 6 (elovl6), fatty acid binding protein 1 (fabp1),* and *sterol regulatory binding protein 1c (srebp1c)* were also downregulated in the maqui-supplemented mice (Figure 4). Furthermore, maqui administration also reduced the expression of *carbohydrate-responsive element-binding protein beta (chrebpb),* a transcriptional factor that regulates hepatic lipogenesis.

## 4. Discussion

Our study shows the effect of dietary supplementation with a lyophilized maqui berry preparation on the progression of diet-induced obesity in mice subjected to a HFD for 16 weeks. In the liver, dietary maqui supplementation decreases the amount of TG and ameliorates the hepatic steatosis caused by a HFD. It is well-described that in fatty liver disease there is an increase in hepatic lipid accumulation together with an increase in PPARa activity, as well as an upregulation of gluconeogenic, beta-oxidative, and ketogenic gene expression [38]. In our experimental model, the dietary maqui supplementation reversed this metabolic profile. The dietary maqui-supplemented animals exhibited an improvement in the hepatic lipid content together with a downregulation of key genes from fatty acid oxidation, gluconeogenesis, and de novo lipogenesis pathways. Moreover, our results point out the upregulation of the SMILE mRNA levels as part of the molecular mechanism underlying the metabolic profile observed in the livers of HFDM mice.

**Figure 5:**
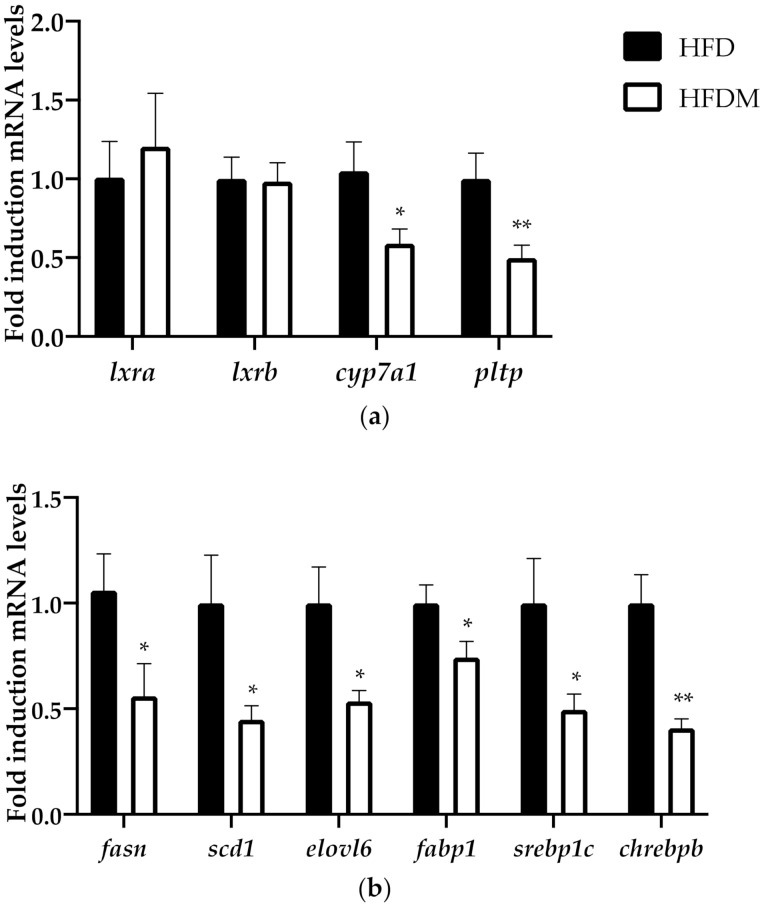
Maqui downregulated the LXR-target genes, including the expression of de novo lipogenesis genes. (**a**) Relative hepatic mRNA levels of *liver x receptor alpha (lxra), liver x receptor beta (lxrb), cholesterol 7 alpha-hydroxylase (cyp7a1),* and *phospholipid transfer protein (pltp)* were measured via qRT-PCR in HFD (*n* = 9) and HFDM (*n* = 14). (**b**) Relative hepatic mRNA levels of *liver fatty acid synthase (fasn), stearoyl-CoA desaturase-1 (scd1), fatty acid elongase 6 (elovl6), fatty acid binding protein 1 (fabp1), sterol regulatory binding protein 1c (srebp1c),* and *carbohydrate-responsive element-binding protein beta (chrebpb)* were measured using qPCR in HFD (*n* = 9) and HFDM (*n* = 14) animals. Bars represent the relative mRNA levels in the HFD animals, which was the control group and was thus assigned to an arbitrary value of 1, and in the HFDM group. Data are presented as the mean ± SEM. * *p* < 0.05 versus the HFD group; ** *p* < 0.01 versus the HFD group.

SMILE is a nuclear receptor family corepressor that has been implied in the maintenance of lipid homeostasis by regulating SREBP1c and LXR activities. On one side, it has been described that the activity of the lipogenic transcription factor SREBP-1c is induced by SMILE because it inhibits the insulin-induced gene 1 (Insig) protein and activates SREBP1c maturation [39]. On the other side, SMILE inhibits LXR agonist-induced lipogenic gene expression, thus reducing de novo lipogenesis [29]. Together, SMILE-mediated activation of SREBP-1c and repression of LXR has been proposed as a regulatory mechanism to maintain hepatic lipid synthesis in response to nutrient availability [39]. In our experimental approach, dietary maqui supplementation induces *smile* (Figure 4), which in turn would block LXR transcriptional activity as is shown by the downregulation of the LXR-target genes *cyp7a1* and *pltp,* as well as the mRNA levels of several lipogenic genes including *srebp1c* (Figure 5). Besides the classical lipogenic genes, maqui dietary supplementation also reduces the mRNA levels of *chrebpb*. ChREBPb is a transcriptional factor initially identified as a glucose-responsive factor that has recently been described as essential for fructose-induced lipogenesis both in the small intestine and liver [40,41,42,43]. The reduced expression of *chrebpb* in the liver of HFDM (Figure 5b), together with the other genes analyzed, would contribute to diminishing the de novo lipogenesis rate in maqui-supplemented mice. High fructose diets are considered pernicious as they induce lipogenesis, hepatic steatosis, and, finally, the development of insulin resistance and metabolic syndrome [43,44,45]. Under maqui supplementation, the lack of induction in the hepatic expression of *chrebpb* suggests that fructose from maqui does not promote the lipogenic pathway. These results reinforce the idea that the metabolic effects of fructose are not the same when fructose is consumed within its natural source or as an added sugar. It is well-described that the overconsumption of sugar-sweetened beverages or foods, or high-fructose diets, leads to adverse effects on health but, on the other hand, it is accepted that fruit intake is protective for human health. Evidence about the effects of consuming natural sources of free sugars, such as fruits, is still scarce [46,47].

Besides de novo lipogenesis, SMILE also regulates the gluconeogenic pathway. Our results showed that blood fasting glucose levels in HFDM mice were lower than in HFD, thus indicating less hepatic glucose production in maqui-supplemented mice. This lower production of glucose in the liver of HFDM mice correlates with the upregulation of *smile*, the downregulation of *pgc1a* and the reduction in *g6Pase* expression. A recent study demonstrated that SMILE is an insulin-inducible corepressor that decreases *pgc1a* expression and the stimulatory effect of PGC1a on hepatic gluconeogenesis [37]. This effect is produced by the interaction and disruption of the CREB/CRTC2 complex by SMILE that results in a significant inhibition of CRTC2-induced PGC1a expression [48]. Furthermore, SMILE also inhibited CREB/CRTC2-induced *pepck* and *g6Pase* gene expression by a direct transcriptional repression of these genes [48]. Although no changes were observed in the *pepck* mRNA levels, globally our data suggest that maqui diminishes the gluconeogenic pathway in the livers of diet-induced obese mice.

Another pathway closely related to lipid homeostasis is fatty acid oxidation. It is well-known that a tight regulation of hepatic fatty acid oxidation is essential for maintaining an energy balance. Moreover, liver steatosis susceptibility has been associated with PPARa dysfunction [49]. Considering that PPARa is a key regulator of hepatic fatty acid oxidation, we analyzed the expression of PPARa and the fatty acid oxidation-related PPAR alpha target genes in the liver. Our analysis showed a decrease in fatty acid oxidation gene expression in the maqui-supplemented mice. According to the putative requirement of fatty acid oxidation in gluconeogenesis, these data reinforce the lower gluconeogenic rate detected in the livers of HFDM mice.

Given the role of SMILE as a corepressor of the nuclear receptor family, the inhibition of the PPARa transcriptional activity could be attributed to SMILE. It has been described that SMILE blocks the activity of several other nuclear receptors such as PPAR gamma, the estrogen receptor, the androgen receptor, or LXR, among others [28,50,51,52]. Although no experimental data are available about its effect on PPARa activity, the inhibition of PPARa transcriptional activity in HFDM mice could be attributed to SMILE. Furthermore, the observed downregulation of PGC1a may also contribute to the decreased oxidation of fatty acids in HFDM mice.

The remaining question is what component or components of maqui are responsible for its metabolic effects. Ursolic acid is a naturally occurring pentacyclic triterpenoid carboxyl acid with a wide range of biological activities, including hepatoprotective and hypolipidemic effects, both of which have the capacity to reduce hepatic lipid accumulation [50,53]. It has been shown that ursolic acid significantly decreased the mRNA and protein expression of LXRa target lipogenic genes including SREBP1c. The proposed mechanism underlying the effects of ursolic acid is an AMPK-dependent increase in the corepressor SMILE that would antagonize LXRa [50]. Ursolic acid is present in the dichloromethane extract of the leaves of *Aristotelia chilensis* [54], thus suggesting that it may also be present in the lyophilizate maqui used in this work. Besides the anthocyanin profile described in the lyophilizate maqui [23], which surely explains part of the beneficial effects attributed to maqui, ursolic acid, if present, may contribute significatively to the hypolipidemic effects observed in maqui dietary supplemented mice. We aim to test this hypothesis in future work.

## 5. Conclusions

**Figure 6:**
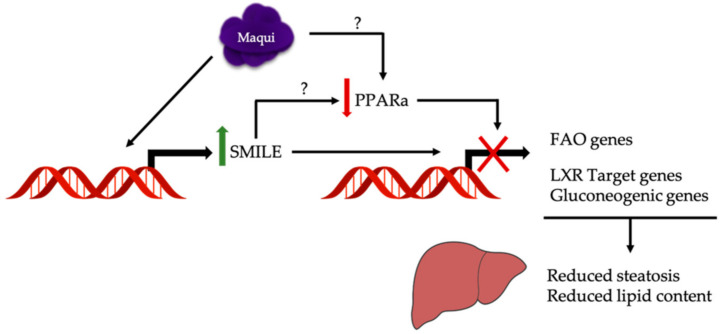
Maqui transcriptionally regulates a metabolic response to counteract hepatic steatosis and increased lipid content caused by a HFD. The hepatic metabolic changes observed in maqui-supplemented mice include a downregulation of the lipogenic LXR target gene and fatty acid oxidation and gluconeogenic gene expression together with an increased expression of SMILE, a corepressor of the nuclear receptor family. The upregulation of SMILE may be the mechanism underlying the downregulation of PPARa transcriptional activity but a direct effect of maqui on PPARa expression/activity cannot be discarded.

Our data indicated that maqui supplementation transcriptionally regulates lipid handling in the liver in order to counteract the metabolic impact of HFD, resulting in decreased hepatic steatosis (Figure 6). Changes in the hepatic metabolic profile include a downregulation of lipogenic LXR target genes and of fatty acid oxidation gene expression together with an increased expression of SMILE, a corepressor of the nuclear receptor family.

In conclusion, our data reinforce the use of anthocyanidin-enriched foods as a potential strategy to prevent or treat obesity-related diseases and identify maqui as a putative functional fruit to counteract obesity and its metabolic complications. The data presented in this manuscript support the inclusion of maqui in the diet of obese individuals.

## Data Availability

The data presented in this study are available on request from the corresponding author.

## Supplementary Materials

The following are available online at https://www.mdpi.com/article/10.3390/antiox10050637/s1, Table S1: Sequences of the primers used in SYBR green assays and references of the probes used in TaqMan assays. Figure S1: Stability of the Housekeeping genes.
