# Supplementary material for: Lyophilized Maqui (Aristotelia chilensis) Berry Administration Suppresses High-Fat Diet-Induced Liver Lipogenesis through the Induction of the Nuclear Corepressor SMILE
Source: Antioxidants (Basel). 2021 Apr 21;10(5):637. doi: 10.3390/antiox10050637 (PMC8143281; doi:10.3390/antiox10050637)

Table S1: Sequences of the primers used in SYBR Green assays and references of the probes used in TaqMan assays. The NCBI accession numbers have been included.

**Gene**	**TaqMan Probe**	
18s	4310893E – 1510059	
mPpara	Mm00440939_m1	
mFabp1	Mm00444340_m1	
	**Primer Forward (SBYR green)**	**Primer Reverse (SBYR green)**
mB2M<br>NM_009735.3	F-5`-ACTGATACATACGCCTGCAGAGTT -3`	R-5`-TCACATGTCTCGATCCCAGTAGA -3`
mCPT1a<br>NM_013495.2	F-5`- AGAATCTCATTGGCCACCAG -3`	R-5`- CAGGGTCTCACTCTCCTTGC -3`
mEhhadh<br>NM_023737.3	F-5`-AATACAGCGATACCAGAAGCC -3`	R-5`-ATTCCCAGCATCACTTCCG -3`
mG6Pase<br>NM_008061.4	F-5`- CAGTGGTCGGAGACTGGTTC-3`	R-5`- GTCCAGGACCCACCAATACG-3`
mPepck<br>NM_011044.2	F-5`- CTTTGGTGGCCGTAGACCTG-3`	R-5`- GATGATCTTGCCCTTGTGTTCTG-3`
mSmile<br>NM_145151.3	F-5`- GGATACAGCTGTTGGGGCA -3`	R-5`- CAGCAGAAGCCCTGATATTACCT -3`
mPgc1a<br>NM_008904.2	F- 5′-AACCACACCCACAGGATCAGA-3′	R- 5′-CTCTTCGCTTTATTGCTCCATGA-3′
mLxra<br>NM_001177730.1	F-5`- CAGGGTGAGGAGAGGAAGGA -3`	R-5`- ACATCTCTTCCTGGAGCCCT -3`
mLxrb<br>NM_001285517.1	F-5`-GCGGGGCCTGGAACAA -3`	R-5`-GAACCATTCCCAGGCACG -3`
mCyp7a1<br>NM_007824.3	F-5`-TTGATCTGGGGGATTGCTGT -3`	R-5`-GGATCCGAACTTCAGAGCACA -3`

mPltp<br><br>NM_011125.2	F-5`-CATCACCATCCCAGACGTGTA -3`	R-5`- CAGATCTTGGTCTGGCTGGAA-3`
mFasn<br><br>NM_007988.3	F-5`-GCTGCGGAAACTTCAGGAAAT -3`	R-5`-AGAGACGTGTCACTCCTGGACTT -3`
mScd1<br><br>NM_009127.4	F-5`CTTGGGATCTTCCTTATCATT- -3`	R-5`-GATCTCGGGCCCATTCG -3`
mElolv6<br><br>NM_130450.2	F-5`-TGCAGGAAAACTGGAAGAAGTCT -3`	R-5`-ATGCCGACCACCAAAGATAAA -3`
mSrebp1c<br><br>NM_001358314.1	F-5`-GGAGCCATGGATTGCACATT-3`	R-5`-GGCCCGGGAAGTCACTGT-3`
mChrebpb<br><br>NM_001359237.1	F-5`- TCTGCAGATCGGTGGAG-3`	R-5`-CTTGTCCCGGCATAGCAAC -3`

Figure S1: Stability of the Housekeeping genes. Hepatic mRNA relative levels of *18s* and *B2m* were measured by qRT-PCR in HFD (n=9) and HFDM (n=14). Bars represent the mRNA relative levels in the HFD animals, considered the control group and assigned to an arbitrary value of 1, and in the HFDM. Data are presented as the mean ± SEM.

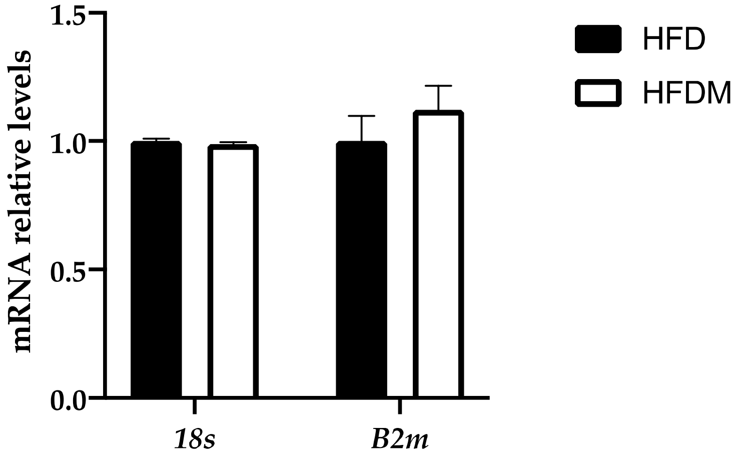

Supplement: Supplementary file 1 [file antioxidants-10-00637-s001.zip › antioxidants-1167597-supplementary.pdf]
